# Screening competition and cross-feeding interactions during utilization of human milk oligosaccharides by gut microbes

**DOI:** 10.20517/mrr.2023.61

**Published:** 2024-01-01

**Authors:** Romina Díaz, Daniel Garrido

**Affiliations:** Department of Chemical and Bioprocess Engineering, School of Engineering, Pontificia Universidad Catolica de Chile, Santiago 7820436, Chile.

**Keywords:** *Bifidobacterium*, cross-feeding interactions, HMO, infant gut microbiome

## Abstract

**Background:** The infant gut microbiome is a complex community that influences short- and long-term health. Its assembly and composition are governed by variables such as the feeding type. Breast milk provides infants an important supply of human milk oligosaccharides (HMO), a broad family of carbohydrates comprising neutral, fucosylated, and sialylated molecules. There is a positive association between HMOs and the overrepresentation of *Bifidobacterium* species in the infant gut, which is sustained by multiple molecular determinants present in the genomes of these species. Infant-gut-associated *Bifidobacterium* species usually share a similar niche and display similar HMO inclinations, suggesting they compete for these resources. There is also strong evidence of cross-feeding interactions between HMO-derived molecules and bifidobacteria.

**Methods:** In this study, we screened for unidirectional and bidirectional interactions between *Bifidobacterium* and other species using individual HMO. *Bifidobacterium bifidum* and *Bacteroides thetaiotaomicron* increased the growth of several other species when their supernatants were used, probably mediated by the partial degradation of HMO. In contrast, *Bifidobacterium longum* subsp. *infantis*. supernatants did not exhibit positive growth.

**Results:** *Bifidobacterium* species compete for lacto-*N*-tetraose, which is associated with reduced bidirectional growth. The outcome of these interactions was HMO-dependent, in which the two species could compete for one substrate but cross-feed on another. 2’-fucosyllactose and lacto-*N*-neotetraose are associated with several positive interactions that generally originate from the partial degradation of these HMOs.

**Conclusion:** This study presents evidence for complex interactions during HMO utilization, which can be cooperative or competitive, depending on the nature of the HMO. This information could be useful for understanding how breast milk supports the growth of some *Bifidobacterium* species, shaping the ecology of this important microbial community.

## INTRODUCTION

The infant gut microbiome represents a complex community of microorganisms that provide essential functions and metabolites to the host. The composition of the gut microbiome dynamically changes during the first year of life and is modulated by various factors, including gestational age^[1]^, delivery mode^[2]^, antibiotic use^[3]^, and infant feeding^[4]^. Considering the factors dictating infant gut microbiome composition, it is distinguishable from the adult gut microbiome as it transitions to a mature state after the introduction of solid foods^[5,6]^.

Diet is one of the primary factors that influence the gut microbiome. Human milk contains infant’s nutrients and bioactive components that support appropriate growth, digestibility, and tolerance^[7,8]^. Breastfeeding has a distinctive protective role since breastfed infants have a lower incidence of diarrhea, allergies, and inflammatory diseases, contributing additional benefits to immune system development^[9-11]^. Breast milk contains high concentrations of free human milk oligosaccharides (HMOs). HMOs are unconjugated glycans with a lactose core and chain lengths varying from 3 to 15 monosaccharide building blocks [glucose, galactose, fucose, *N*-acetylglucosamine (GlcNAc), *N*-acetylneuraminic acid (NeuAc), or sialic acid]^[8,12]^. Abundant and characteristic HMOs include 2’-fucosyllactose (2’-FL), lacto-*N*-tetraose (LNT), 6’-sialyllactose (6’-SL), and lacto-*N*-neotetraose (LNnT).

Importantly, HMOs are usually fermented by beneficial *Bifidobacterium* species^[13]^. The establishment of bifidobacteria in the infant gut is supported by the abundance of multiple carbohydrate utilization genes and specific gene clusters in these species, facilitating the fermentation of multiple HMO types^[14-17]^. The fermentative metabolism of *Bifidobacterium* generates short-chain fatty acids (SCFA), primarily acetate and lactate (organic acid)^[18]^.

Ecological relationships, vary from competition to mutualism and syntropy and are important determinants of community composition and function^[19]^. An important driver of these relationships is the metabolic potential of the interacting members of a network, which can result in competition for resources or cross-feeding for mutual benefit^[20,21]^. Interspecies relationships in the human gut microbiome are context-dependent, and multiple factors may influence specific connections under a given condition. Therefore, knowledge of interspecies metabolic interactions in the presence of several other species with beneficial or competitive roles is crucial for understanding and predicting the function and stability of microbial communities^[22]^.

The utilization of HMOs by *Bifidobacterium* has been studied at the single-species level using conventional methods^[23-25]^. Nevertheless, there are few examples of microbial interactions mediated by HMOs that consider cooperation and competition, especially when using a different method (transwell plates). Nishiyama *et al*. investigated the role of two extracellular sialidases in the cross-feeding between *B. bifidum* and *B. breve* strains using 6’-SL. The results revealed that consuming 6’-SL by *B. bifidum* released sialic acid, which allowed the growth of *B. breve*^[26]^. Ojima *et al*. applied the assembly theory to a community of four representative infant-gut-associated *Bifidobacterium* species that employed various strategies for HMO consumption, showing that arrival order and sugar consumption phenotypes significantly affected community establishment^[27]^. Some HMO fermenters, such as *Bifidobacterium* spp., release acetate and lactate as final products in the environment. Their fermentation efficiency is increased by lactate or acetate fermenters such as *Eubacterium hallii*, *Anaerostipes caccae*, and butyrate producers, highlighting the important role of *Bifidobacterium* in cross-feeding interactions^[28]^.

In this study, we aimed to explore microbial interactions between *Bifidobacterium* and other infant gut microbes, such as *Bacteroides* and *Lachnoclostridium*, using a single HMO to study the differences in interaction outcomes. The novel aspects that we aimed to bring are (a) microbial interactions were different depending on the HMO; (b) the use of Transwell plates to study HMO utilization to determine metabolite sharing; and (c) the different interactions, such as *B. bifidum* and *B. thetaiotaomicron*, which were much higher than that of *B. infantis*.

## MATERIALS AND METHODS

### Bacterial strains and cultured media

Twelve strains belonging to four different phyla (*Actinobacteria*, *Bacteroidetes, Firmicutes,* and *Proteobacteria*) were included in this study. The strains from BEI Resources, UC Davis Culture Collection, and Chilean isolates are summarized in Table 1. For routine experiments, the microorganisms were cultured in their respective media [Table 1]. Luria-Bertani medium (LB; Becton, Dickinson, Franklin Lakes, NJ) was used directly, while according to the case, reinforced clostridium broth (RCM; Becton, Dickinson, Franklin Lakes, NJ) and Man-Rogosa-Sharpe broth (MRS; Becton, Dickinson, Franklin Lakes, NJ) were supplemented with 0.05% w/v of L-cysteine-HCl (Sigma-Aldrich, St. Louis, MO, USA). The cultures were incubated at 37 °C for 24-48 h in an anaerobic jar (Anaerocult, Merck, Darmstadt, Germany) with anaerobic packs (Gaspak EM; Becton-Dickinson, Franklin Lakes, NJ, USA).

**Table 1:** Description of strains, genomic features, and media used in this study

**Phylum, species, and strain**	**Genome size (Mbp).** **%GC**	**SCFA and organic acids production**	**Culture medium**	**Source**	**Ref.**
*Actinobacteria:*					
*Bifidobacterium infantis* ATCC 15697	2.83; 59.86%	Acetic and lactic acid	MRS-Cys	UC Davis collection	[18]
*Bifidobacterium bifidum* JCM 1254	2.18; 62.63%	Acetic and lactic acid	MRS-Cys	UC Davis collection	[18]
*Bifidobacterium breve* I1^#^	2.48; 58.67%	N.D	MRS-Cys	Chilean isolated	N.D
*Bifidobacterium longum* M12^#^	2.25; 59.79%	Acetic and lactic acid	MRS-Cys	Chilean isolated	[32]
*Bifidobacterium longum* D4^#^	2.31; 59.78%	Acetic and lactic acid	MRS-Cys	Chilean isolated	[32]
*Bifidobacterium longum* SC664	2.48; 59.74%	Acetic and lactic acid	MRS-Cys	UC Davis collection	[18]
*Bacteroidetes:*					
*Bacteroides thetaiotaomicron* VPI-5482	6.29; 42.86%	N.D	RCM-Cys	UC Davis collection	[33]
*Bacteroides vulgatus* S1^#^	5.00; 42.31%	N.D	RCM-Cys	Chilean isolated	N.D
*Firmicutes:*					
*Lachnoclostridium symbiosum* WAL 14673	5.31; 47.73%	5.31; 47.73%	RCM	BEI resources	[49]
*Streptococcus thermophilus* LM8^#^	2.10; 39.96%	Lactic acid	MRS	Chilean isolated	[50]
*Enterococcus faecalis* H1^#^	4.76; 38.50%	N.D	RCM	Chilean isolated	[51]
*Proteobacteria:*					
*Escherichia coli* K12 MG1655	4.64; 50.79%	Acetic and lactic acid	LB	BEI resources	[52]

The table shows the general characteristics of the 12 strains used in the unidirectional and bidirectional assays. ^#^Chilean strains. Cys: Cysteine; LB: Luria-Bertani medium; MRS: Man-Rogosa-Sharpe medium; N.D: not determined; RCM: reinforced clostridium medium; SCFA: short-chain fatty acids.

### Monoculture and co-culture experiments

The optimized mZMB formulation was used for bacterial growth^[29]^ [Supplementary Material]. This culture medium allowed the growth of multiple gut microbiome species. Before inoculation, the microorganisms were grown in a monoculture (mZMB supplemented with 20 g/L lactose) in an anaerobic jar (Anaerocult, Merck, Darmstadt, Germany) with anaerobic packs (Gaspak EM, Becton-Dickinson, Franklin Lakes, NJ, USA) for 48 h at 37 °C. For the unidirectional experiments, a microplate reader (Tecan Trading AG, Austria) was placed in the chamber under anaerobic conditions. The mZMB medium was supplemented with each HMO type (10 g/L) in 96-well microplates (Corning Inc., USA) for unidirectional assays and Tissue Culture Plate Inserts (Transwell plates) (JetBiofil, China) were used for bidirectional assays.

### Unidirectional assays

Three early infant gut colonizers were designated primary degraders and used for unidirectional assays. We followed the experimental design described by de Vos *et al*. (2017) to assess the cross-feeding potential of 12 strains. Briefly, cell-free supernatants (4 mL) were generated by centrifugation at 9,000 rpm, followed by sterile filtration (0.22 µm filter) of each primary degrader grown anaerobically for 24 h in mZMB + 1% (w/v) HMO (2’-FL, LNT, and LNnT)^[30]^. The HMOs were provided by Glycom (Denmark). New media reconstitution was prepared by adding mZMB without a carbon source to each cell-free supernatant (1:1). In addition, secondary degraders were cultured in Eppendorf tubes in their respective media and washed with mZMB without a carbon source before inoculation. The reconstituted medium was used as a new medium to culture microorganisms. Incubations were conducted in 96 well plates (inoculation at 10%, according to anaerobic conditions) at 37 °C for 48 h under anaerobic conditions (nitrogen 99% purity) in an anaerobic chamber (Sheldon Manufacturing INC, Bactronez-2 Anaerobic Chamber Workstation, Cornelius, OR, USA). Anaerobic growth was monitored every 30 min using a microplate spectrophotometer at 620 nm (Tecan Infinite F50, Männedorf, Switzerland). The initial OD_620_ value for each microorganism was removed from the subsequent measurements. The experiments were performed in triplicate. mZMB + 2% (w/v) lactose was used as a positive control.

### Bidirectional assays

The bacterial strains were cultured in Tissue Culture Plate Inserts (Transwell plates) (JetBiofil, Beijing, China). The microorganism designated as the primary degrader was always cultured in the lower well, whereas the other microorganism designated as the secondary degrader was cultured in the upper insert. The strains were separated by a permeable membrane in the insert (0.1 µm), allowing small carbohydrates and metabolites to be passed. Co-cultures were performed in 250 L of mZMB without a carbon source in the upper insert, and 1 mL of mZMB with lactose or HMO in the lower well. Mono- and co-culture conditions were tested in duplicate (in the lower well) using 2% w/v lactose (Sigma-Aldrich) and 1% w/v of each HMO (2’-FL, 3’-SL, and LNT) (Glycom, Denmark) as the sole carbon source. The strains were reactivated in the respective media for 48 h, centrifuged at 12,000 rpm for 1 min, and washed with mZMB without a carbon source. Inoculation was performed at 5% w/v in an anaerobic jar (Anaerocult; Merck, Darmstadt, Germany) with anaerobic packs (Gaspak EM; Becton-Dickinson, Franklin Lakes, NJ, USA) at 37 °C for 24 and 48 h. OD_620_ was measured at 0, 24, and 48 h by resuspending each well’s content and transferring 200 L to a new 96-well plate. Measurements were performed using an Infinite M200 PRO spectrophotometer (Tecan Trading AG, Infinite M200 PRO, Männedorf, Switzerland). The measurements obtained at 0 h were removed from other measurements to ensure reliable bacterial growth. The samples were then transferred to Eppendorf tubes and centrifuged at 12,000 rpm for 1 min, and the supernatant was separated from the pellets. Both were stored at -80 °C until use.

### Substrate consumption

HMO consumption was analyzed by thin-layer chromatography (TLC). Galactose, glucose, fucose, lactose, 2’-FL, LNT, and LNnT were standard. The polar stationary phase was a silica gel plate adhered to a solid surface (Fluka Silica Gel 02549-20EA). The mobile phase was a solution of N-butanol, acetic acid (99% purity), and chromatography-grade water at 2:1:1 (v/v). A solution of 0.5% α-naphthol, 5% sulfuric acid, and ethanol was used as the solvent to visualize the spots. In addition, it is necessary to incubate the plate with heat for color development^[31]^.

### Statistical analysis

Statistical analysis was performed using technical triplicate or duplicate data. Graphs were created using PRISM v 9.4.1 (GraphPad, La Jolla, CA). Univariate analysis of variance or two-way analysis of variance (ANOVA) and Tukey’s test were performed to determine the significant differences between the groups for growth, oligosaccharide consumption, and metabolite production. Statistical significance was set at *P* ≤ 0.05.

## RESULTS

### Monoculture growth of infant gut-associated strains used in interaction assays

The strains used in the present study are listed in Table 1. The table includes the infant’s gut microbiome-associated *Bifidobacterium* species, two *Bacteroides* species, a butyrate producer, a lactic acid bacterium, *Enterococcus faecalis,* and *Escherichia coli*. Table 2 summarizes the growth of each bacterium (monoculture) on 2’-FL, LNT, LNnT, 3’-SL, and lactose (positive control). As expected, *B. infantis* and *B. bifidum* displayed good growth on these substrates, similar to that of *Bacteroides vulgatus*. *B. breve* I1 grew only on LNT and LNnT. *B. longum* M12 grew on 2’-FL, as previously reported^[32]^. *E. coli*, *L. symbiosum*, and *Streptococcus thermophilus* grew only on lactose [Supplementary Data 1]. Based on these results and available public data on the genomic features of the strains, *B. infantis* and *B. bifidum* were considered primary HMO degraders. In addition, *B. thetaiotaomicron* was selected as a weak primary HMO degrader based on a previous study by Marcobal *et al*.^[33]^. Moreover, moderate (++), low (+), and OD_620nm_ < 0.15 HMO were categorized as secondary HMO consumers in the unidirectional assays, which might have the ability to use degradation products released by the first group. Furthermore, *B. vulgatus* S1 was not considered in this study because it is a Chilean strain that requires further genomic analysis and a comprehensive bioinformatics approach. However, this study could be useful in applying this methodology to novel infant gut microbiome strains.

**Table 2:** Growth monoculture profiles of strains used in this study

**Phylum**	**Strain**	**Abbreviation**	**mZMB media**				
			**Lac 2%**	**2’FL 1%**	**LNT 1%**	**LNnT 1%**	**3’SL 1%**
*Actinobacteria*	*B. infantis* ATCC 15697	ATCC 15697	+++	++	+++	++	+
	*B. bifidum* JCM 1254	JCM 1254	+++	+++	+++	+++	++
	*B. breve* I1^#^	I1	+++	-	+++	++	-
	*B. longum* M12^#^	M12	+++	++	++	-	-
	*B. longum* D4^#^	D4	+++	-	++	-	-
	*B. longum* SC664	SC664	+++	-	++	++	-
*Bacteroidetes*	*B. thetaiotaomicron* VPI-5482	VPI-5482	++	+	+	-	-
	*B. vulgatus* S1^#^	S1	+++	+++	+++	+++	+++
*Firmicutes*	*L. symbiosum* WAL 14673	WAL 14673	+++	-	-	-	-
	*S. thermophilus* LM8^#^	LM8	+++	-	+	-	-
	*E. faecalis* H1^#^	H1	++	++	+	-	+
*Proteobacteria*	*E. coli* K12 MG1655	K12	+++	+	+	-	+

The table shows the strain growth profiles of mZMB supplemented with different HMOs as the sole carbon source. The level of growth was classified as follows: - (OD620_nm_ < 0.15); + (0.15 < OD620_nm_ < 0.50); ++ (0.50 < OD620_nm_ < 0.80); +++ (OD620_nm_ > 0.80). mZMB + 2% w/v lactose was used as a positive control. ^#^Chilean strains. HMOs: Human milk oligosaccharides; LNnT: lacto-*N*-neotetraose; LNT: lacto-*N*-tetraose; 2’-FL: 2’-fucosyllactose; 3’-SL: 3’-sialyllactose.

### Cross-feeding interactions in the unidirectional assays

**Figure 1:**
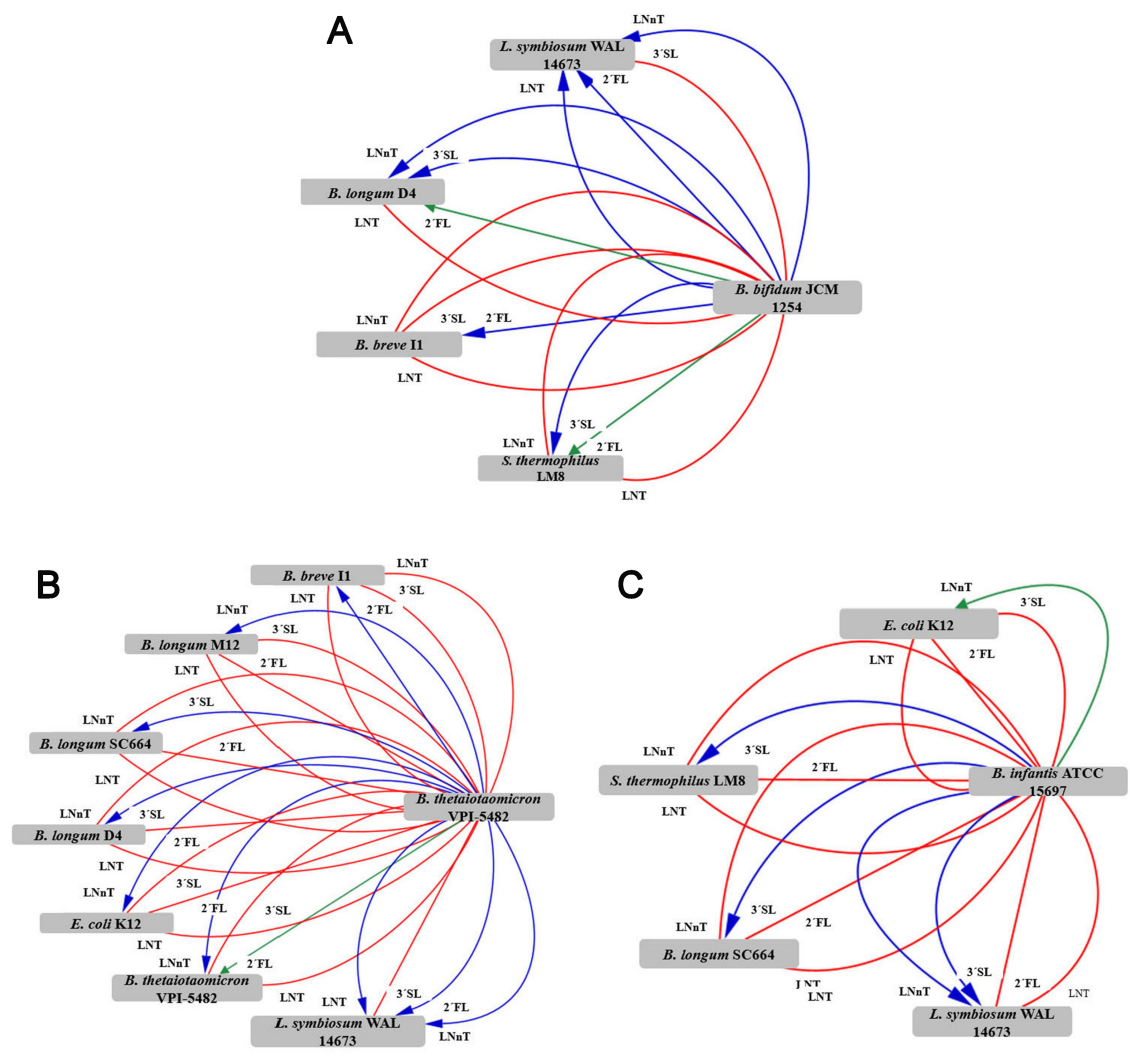
Microbial networks in unidirectional assays. The networks show interactions between primary degraders: (A) *B. bifidum* JCM 1254; (B) *B. thetaiotaomicron* VPI-5482; and (C) *B. infantis* ATCC 15697 and secondary degraders (gray nodes in the network). Interactions in the network were classified as positive, blue arrows (OD620 monoculture < OD620 unidirectional); neutral, green arrows (OD620 monoculture = OD620 unidirectional); or negative, red arrows (OD620 monoculture > OD620 unidirectional) [Supplementary Data 6].

Cell-free supernatants from each primary degrader were reconstituted in HMO-free medium (mZMB) and inoculated with secondary degraders. The spent supernatant of *B. bifidum* (primary degrader) cultured on certain HMOs resulted in positive interactions with *L. symbiosum*, *B. breve*, *B. longum* D4, and *S. thermophilus* [Figure 1A and Supplementary Data 2].

When *B. thetaiotaomicron* VPI-5482 was designated as the primary degrader, several bacteria grew well in the 2’-FL, 3’-SL, LNT, and LNnT supernatants [Figure 1B and Supplementary Data 3]. Moreover, the microbial network generated by *B. infantis* ATCC 15697 as the primary degrader was limited, with some positive interactions, especially involving the 3’-SL supernatant [Figure 1C and Supplementary Data 4]. It is likely that *B. longum* subsp. *infantis* supernatants did not release any degradation products, which is consistent with the mechanism of HMO consumption^[16,34,35]^.

**Figure 2:**
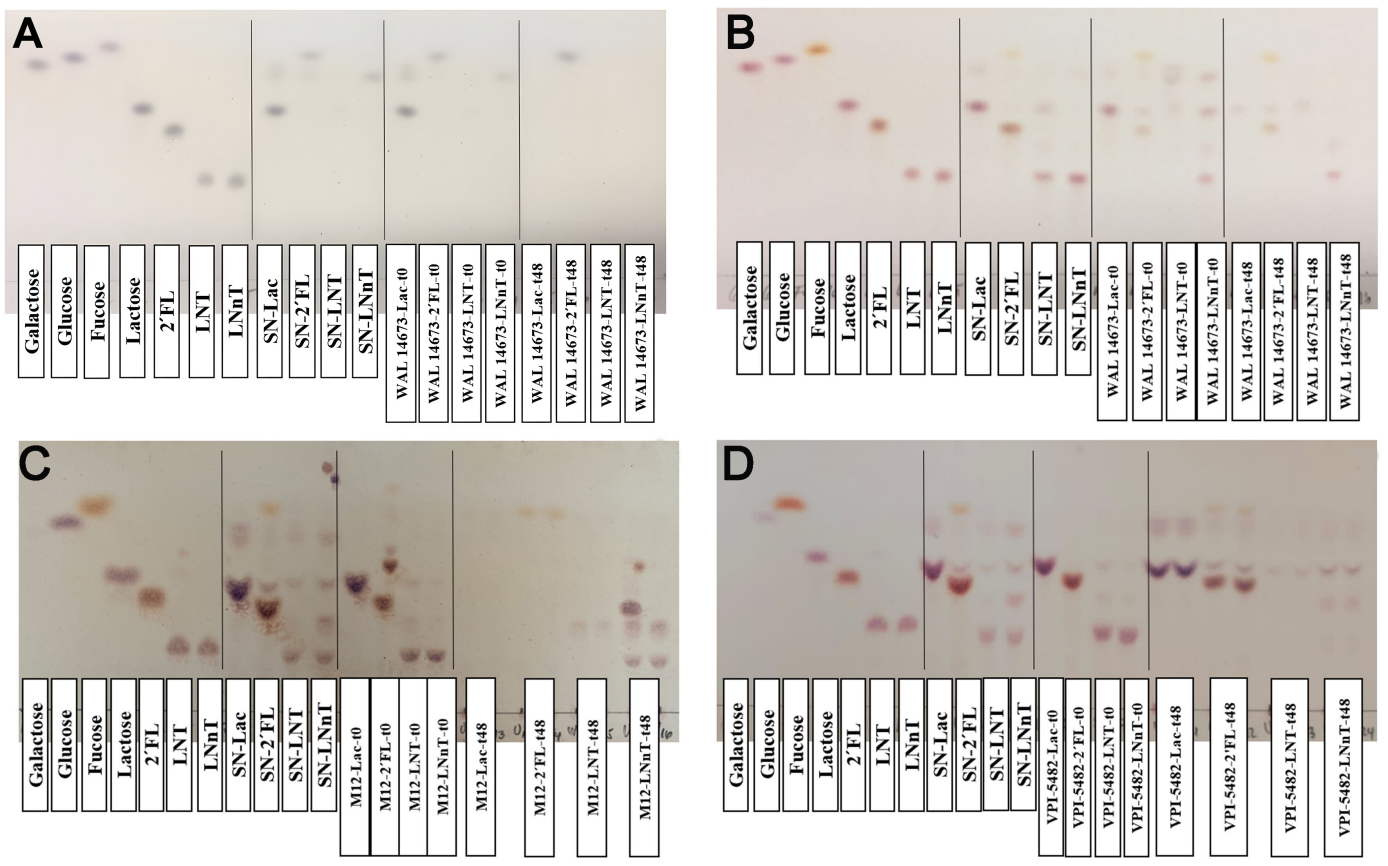
Carbohydrate analysis of unidirectional interactions. TLC plates of secondary degraders: (A) *L. symbiosum* WAL 14673 from *B. bifidum* JCM 1254 supernatants; (B) *L. symbiosum* WAL 14673; (C) *B. longum* M12; and (D) *B. thetaiotaomicron* VPI-5482 (secondary degrader) from *B. thetaiotaomicron* VPI-5482 supernatants. SN: Supernatant obtained from the primary degrader after 24 h of culture; t0: time 0 (secondary degrader inoculation); t48: 48 h of secondary degrader culture.

*L. symbiosum* displayed high growth in the supernatant of 2’-FL, LNT, and LNnT from *B. bifidum*, and it removed lactose and galactose but not fucose, according to the TLC analysis [Figure 2A]. LNT and LNnT supernatants from *B. bifidum* did not contain detectable oligosaccharides [Figure 2A].

The supernatants of this *B. thetaiotaomicron* VPI-5482 displayed multiple degradation products of varying sizes, suggesting that partial HMO utilization by this bacterium released simpler mono- and disaccharides that were available for other microbes. *L. symbiosum* consumed lactose and monosaccharides from LNT and LNnT-derived supernatants [Figure 2B]. *B. longum* M12 removed 2’-FL, lactose, and fucose from *B. thetaiotaomicron* 2’-FL supernatant, and most of the degradation products remained in the LNnT supernatant [Figure 2C]. Most HMOs were consumed when *B. thetaiotaomicron* was inoculated in its own spent supernatant, suggesting that this bacterium has a slow growth rate on HMO, as it can continue using these molecules in a second culture [Figure 2D].

### Competition and cross-feeding interactions detected in bidirectional assays

We later performed paired co-cultures separated by a Transwell membrane to determine possible competition or cooperation among bifidobacterial species in HMO-supplemented media. In this study, some assays have suggested competition between strains, with reduced growth for both species in the co-culture compared with monoculture growth, especially on LNT. In some cases, *B. breve* showed reduced growth, but further analysis is necessary to evaluate 2’-FL consumption by *B. breve* I1 Chilean strain. Based on previous studies, we suggest a bidirectional assay between *B. bifidum* and *B. breve* allowed for cooperation between the strains in 3’-SL-supplemented with vigorous consumption of sialic acid by *B. bifidum* metabolism^[26]^.

**Figure 3:**
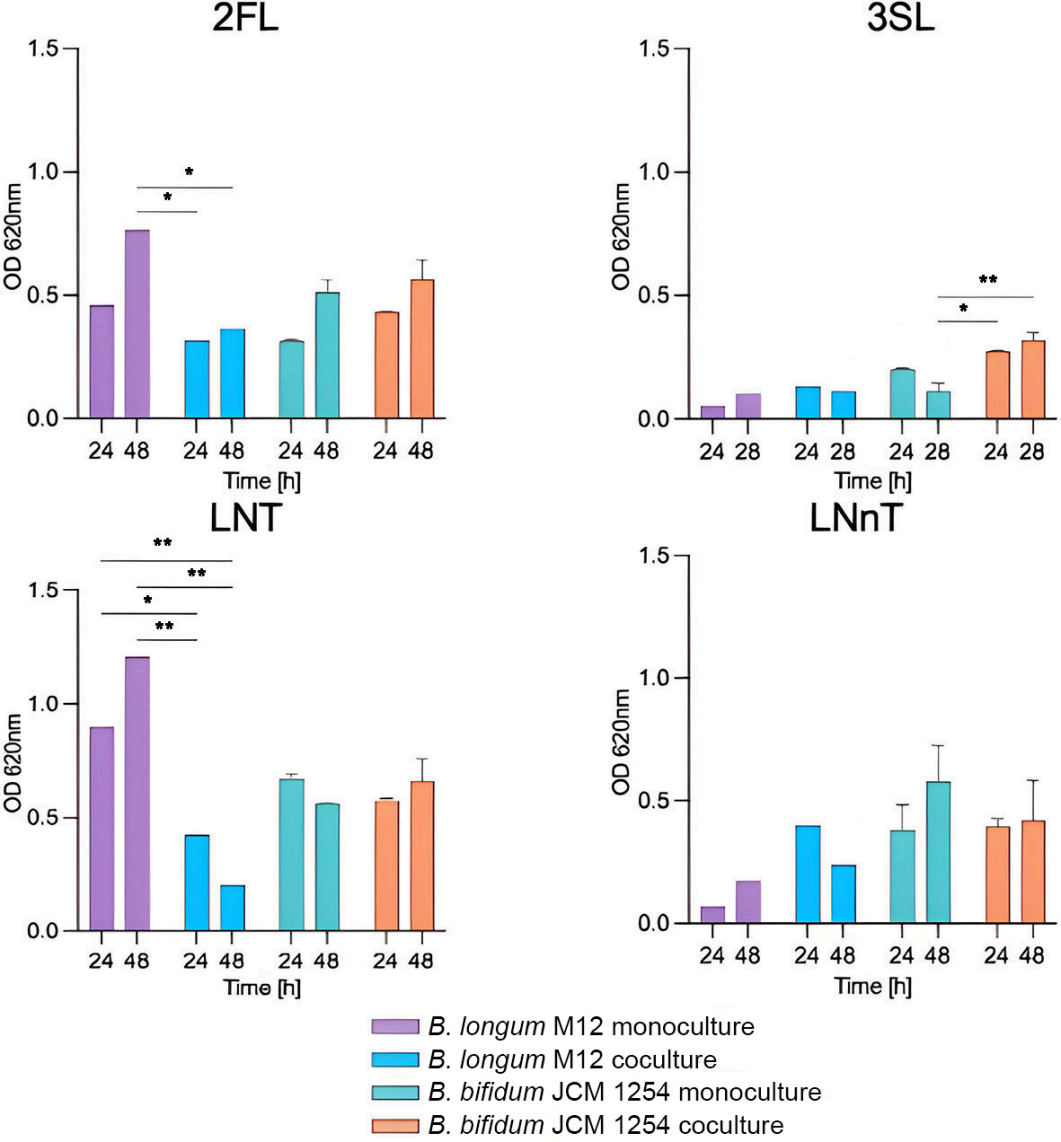
Bidirectional interactions of *B. bifidum* JCM 1254-*B. longum* M12 pair. Growth profiles of bifidobacterial strains in monoculture and co-culture. The growth of the strains in mono- and co-culture was measured at 0, 24, and 48 h. The analysis was performed using technically duplicated data (mean ± SD). Two-way ANOVA and Tukey’s tests were performed to determine significant differences between assays (*P < 0.05, **P < 0.01). *B. bifidum* JCM 1254: primary degrader, *B. longum* M12: secondary degrader. LNnT: Lacto-*N*-neotetraose; LNT: lacto-*N*-tetraose; 2’-FL: 2’-fucosyllactose; 3’-SL: 3’-sialyllactose.

**Figure 4:**
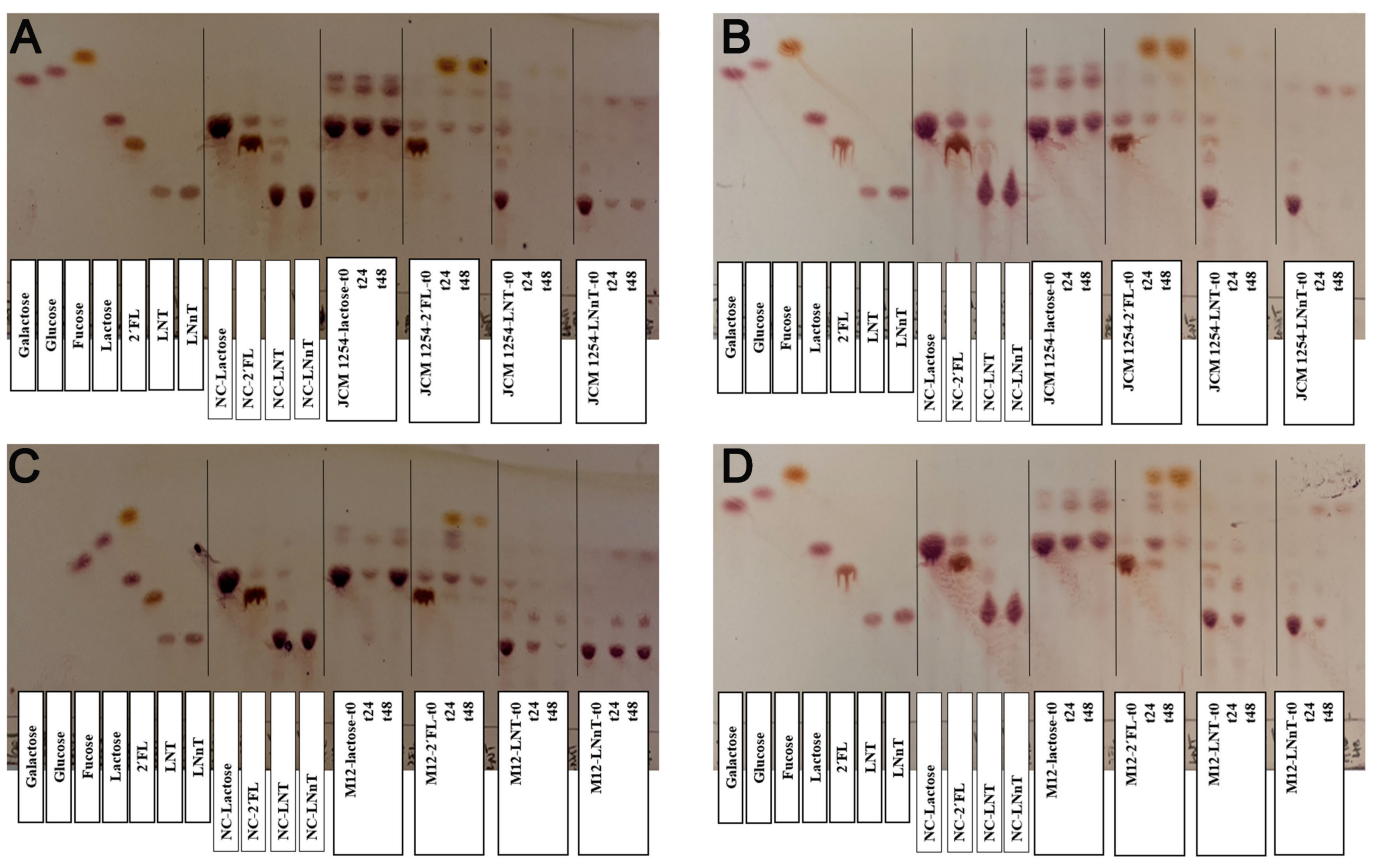
Carbohydrate analysis of *B. bifidum* JCM 1254-*B. longum* M12 interaction. TLC plates display standards, negative controls, and carbohydrate intake at 0, 24, and 48 h of the assay. (A) *B. bifidum* JCM 1254 monoculture; (B) *B. bifidum* JCM 1254 co-culture; (C) *B. longum* M12 monoculture; and (D) *B. longum* M12 co-culture. *B. bifidum* JCM 1254 was cultured in the bottom well, and *B. longum* M12 was cultured in the Transwell upper insert. LNnT: Lacto-*N*-neotetraose; LNT: lacto-*N*-tetraose; TLC: thin-layer chromatography; 2’-FL: 2’-fucosyllactose; 3’-SL: 3’-sialyllactose.

The bidirectional assay used *B. longum* M12 as the secondary degrader and *B. bifidum* as the primary degrader [Figure 3]. Both bacteria consumed 2’-FL and LNT, and the results indicated reduced growth of *B. longum* in the co-culture with *B. bifidum* using these molecules, whereas the latter was not altered [Figure 3]. *B. longum* M12 could not use LNnT but showed a modest increase in growth in the presence of *B. bifidum* in HMO [Supplementary Data 5]. TLC analysis of the 2’-FL supernatants showed no major differences between the mono- and co-cultures, with the accumulation of several degradation products [Figure 4]. The mono- and disaccharide building blocks derived from LNT utilization followed a similar pattern [Figure 4]. The LNnT consumption appears to be faster in the combined growth of *B. bifidum* and *B. longum* than in individual growth [Figure 4].

**Figure 5:**
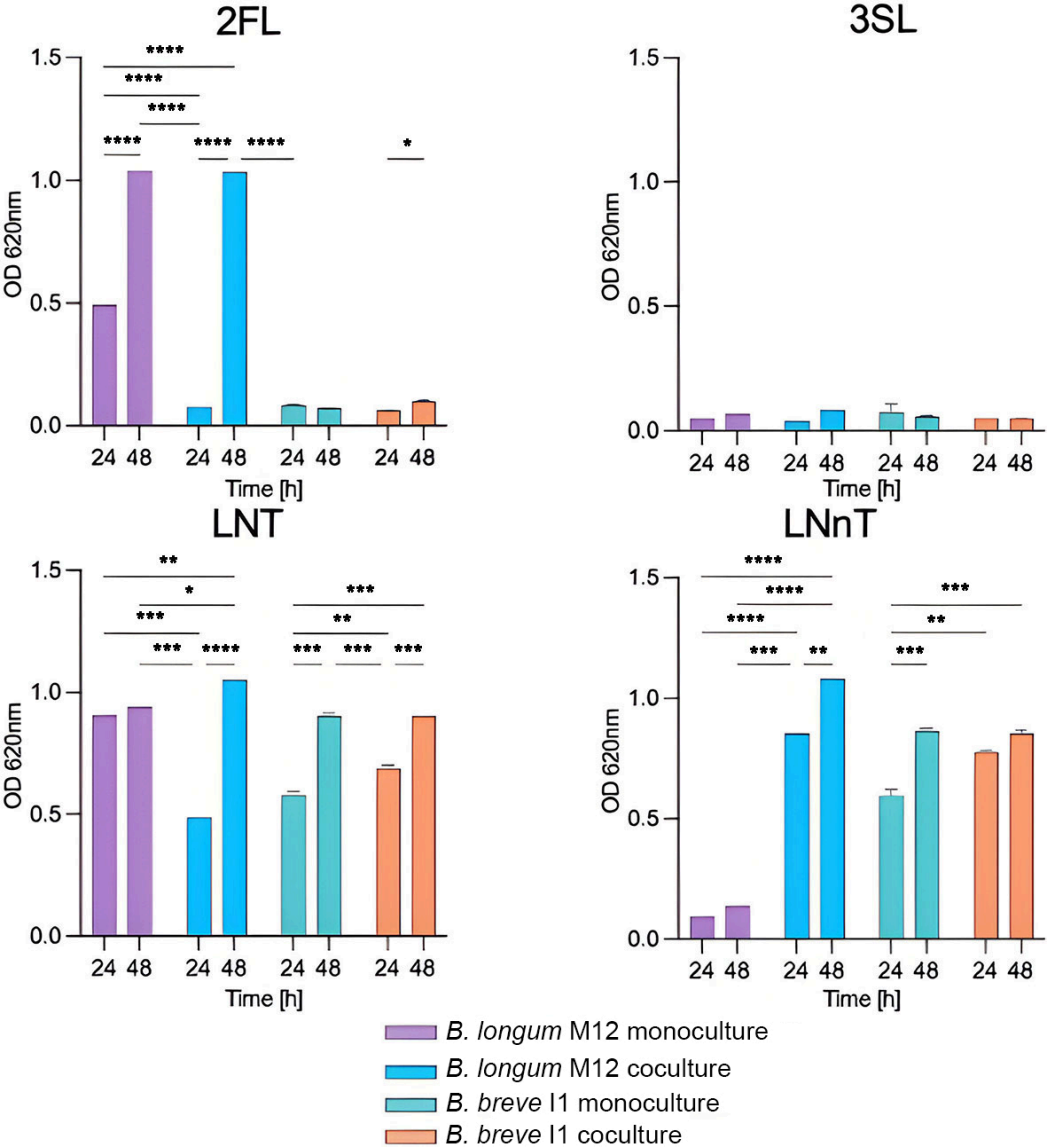
Bidirectional interactions of *B. breve* I1-*B. longum* M12 pair. Growth profiles of bifidobacterial strains in monoculture and co-culture. Growth of strains in mono- and co-cultures at 24 and 48 h (OD_620nm_ at 0 h was the initial inoculation value). The analysis was performed using technically duplicated data (mean ± SD). Two-way ANOVA and Tukey’s tests were performed to determine significant differences between assays (^*^P < 0.05, **P < 0.01, ***P < 0.001, ****P < 0.0001). *B. breve* I1: primary degrader, *B. longum* M12: secondary degrader. ANOVA: Univariate analysis of variance or two-way analysis of variance; LNnT: lacto-*N*-neotetraose; LNT: lacto-*N*-tetraose; 2’-FL: 2’-fucosyllactose; 3’-SL: 3’-sialyllactose.

**Figure 6:**
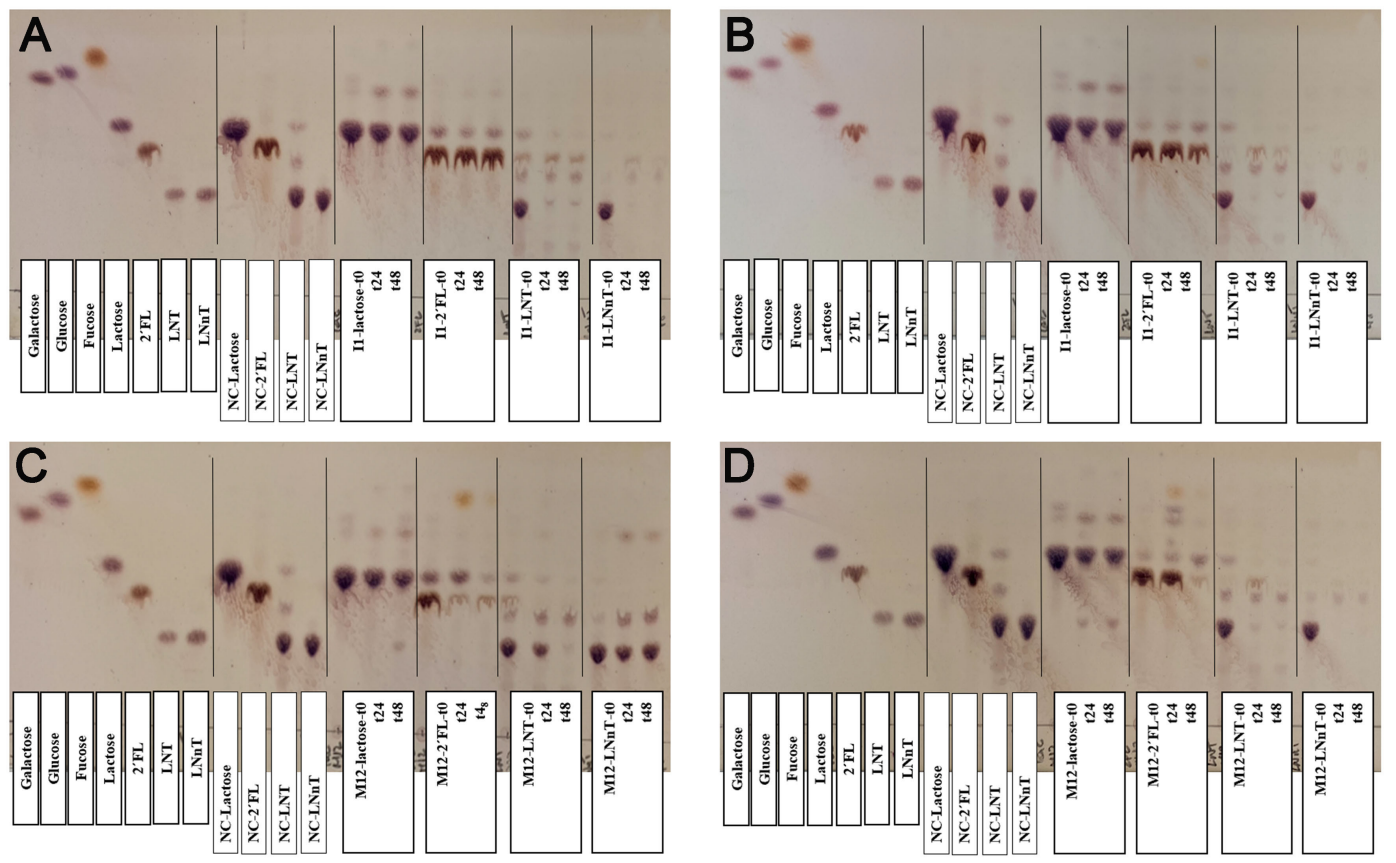
Carbohydrate analysis of *B. breve* I1-*B. longum* M12 interaction. TLC plates display standards, negative controls, and carbohydrate intake at 0, 24, and 48 h of the assay. (A) *B. breve* I1 monoculture; (B) *B. breve* I1 co-culture; (C) *B. longum* M12 monoculture; and (D) *B. longum* M12 co-culture. *B. breve* I1 was cultured in the bottom well, and *B. longum* M12 was cultured in the Transwell upper insert. LNnT: Lacto-*N*-neotetraose; LNT: lacto-*N*-tetraose; TLC: thin-layer chromatography; 2’-FL: 2’-fucosyllactose; 3’-SL: 3’-sialyllactose.

Furthermore, we studied the bidirectional interactions between *B. breve* I1 as the primary degrader and *B. longum* M12 as the secondary degrader. The growth of *B. longum* in 2’-FL did not support the growth of *B. breve* in the co-culture, suggesting vigorous consumption in both approaches [Figure 5]. The final OD values on the LNT showed significant differences between the mono- and co-culture for these microorganisms, which was associated with competition for the substrate [Figure 5]. Interestingly, *B. breve* facilitated *B. longum* on LNnT, significantly increasing its OD without affecting its growth in the co-culture [Figure 5 and Supplementary Data 5]. The TLC results showed increased consumption of LNnT and its degradation products in the co-culture [Figure 6].

## DISCUSSION

Through resource sharing, *Bifidobacterium* species can metabolize fucosylated, sialylated, and neutral HMOs, which may contribute significantly to the assembly of the infant gut microbiome. In this study, we screened for cooperation and competition interactions between infant-gut-associated microorganisms, especially *Bifidobacterium*, using an unconventional method. These results indicate that through simpler experimental analysis, complex interactions of mainly *Bifidobacterium* spp. can compete or cross-feed on specific HMOs.

*B. bifidum* and *B. infantis* are two of the most studied microorganisms regarding the utilization of HMOs. The use of extracellular enzymes and the unique import of a few HMO degradation products in the case of *B. bifidum* markedly differs from the intracellular fermentation of most HMOs by *B. infantis*^[36,37]^. These mechanisms are thought to be due to the higher energy cost of *B. infantis* in terms of multiple glycolytic enzymes, transporters, and primary degrader pathways, compared to the more targeted strategy of *B. bifidum*. These strategies also have consequences at the community level, where these and other results support the partial degradation of HMO by *B. bifidum,* leaving multiple degradation products that can be used by secondary degraders. In contrast, *B. infantis* does not allow major cross-feeding interactions with other microbes^[18,38]^.

Similar to *B. bifidum*, *Bacteroides* are HMO degraders with an extracellular mechanism based on polysaccharide utilization loci (PUL) that induce mucus-utilization genes to consume HMO, resulting in multiple degradation products available for other gut microbes^[39,40]^. Moreover, Marcobal *et al*. studied the genes associated with HMO consumption. The authors found that *Bacteroides thetaiotaomicron* (Bt) possesses a repertoire of glycosyl hydrolases capable of accommodating the structural diversity of HMOs. In addition, the growth of *Bacteroides fragilis*, *B. vulgatus*, and *B. caccae* is comparable to that of Bt^[33]^. Regarding this information, this study was not analyzed in depth using a bioinformatic approach. However, the utilization of HMO by *B. vulgatus* S1 is interesting and requires further investigation. *B. thetaiotaomicron* provides lactose and galactose to *B. longum* derived from 2’-FL, demonstrating cross-feeding, considering that 2’-FL is a recognized HMO-supplemented infant formula^[41]^.

*B. bifidum* metabolism releases mono- and disaccharide building blocks from HMOs to the environment. Therefore, *B. bifidum* can benefit from the growth of other microorganisms by sharing or inhibiting glycan degradation products within the community^[24,42]^. An important observation of this study was the specific network of interactions between the recognized HMO molecules and bifidobacteria. In this regard, LNT could be one of the most effective carbon sources, as almost all infant-associated *Bifidobacterium* strains isolated to date possess the ability to assimilate LNT^[18,43]^. In addition, 2’-FL was revealed to be one of the most abundant HMOs in breast milk containing the secretor status gene (FUT2)^[44]^. Accordingly, *B. bifidum* and *B. longum* showed reduced growth in pairs in the bidirectional assay, suggesting competition for LNT [Figure 3]. LNnT differs from LNT only in the galactose linkage (Galβ1-4 instead of Galβ1-3)^[45]^. *B. bifidum*, *B. breve*, and certain *B. longum* strains can grow on HMO. In agreement with this observation, *B. breve* and *B. bifidum* supported *B. longum* growth in LNnT via degradation products. *B. longum* and *B. breve* are regularly found as the dominant species in infant stools, even though they demonstrate minimal growth in HMO *in vitro*^[25,46]^. Their abundance has been attributed to facilitative priority effects and the availability of vacant niches, as determined by the composition of resident gut microbiota^[27,47]^.

*L. symbiosum* is a *Clostridiales* bacterium characterized by the consumption of lactate, succinate, and simple monosaccharide building blocks for butyrate production. This bacterium grew little in each HMO used as the only carbon source, in contrast to other *Clostridiales* species^[48]^. In this study, *L. symbiosum* acted as a secondary degrader, displaying higher growth when using the supernatants of primary degraders, such as *B. thetaiotaomicron* and *B. bifidum*. Interestingly, lactose and galactose were used as HMO-degradation products from primary degraders. Therefore, the catabolism of HMOs by some gut microbes may support the competitiveness of butyrate-producing Clostridiales via enzymatic activity^[48]^.

In conclusion, the simple experimental analysis presented here contributes to our knowledge of HMO-microbe interactions. This demonstrates the potential of symbiotic patterns to establish a more complex trophic network for microbiome assembly with health-promoting effects on the host. In addition, deciphering the interactions between early colonizers of the infant gut and specific HMO types allows for developing products that closely resemble natural infant microbiome interactions.

This study demonstrates that differences in bacterial cultures and the molecular structure of HMO can considerably impact the biological efficacy and catalytic ability of bifidobacteria strains. The specific molecular strategies used by bifidobacterial strains highlight the importance of generating bacterial networks to achieve efficient HMO metabolism.

Overall, we conclude that the genotypes and phenotypes of microorganisms used in unidirectional and bidirectional assays can be used to create a controlled consortium to investigate metabolic interactions and the roles of key species in a determined environment. Additionally, variations in HMO utilization may determine the fitness and performance of the infant gut microbiome.

Our study analyzed competition and cross-feeding interactions using a simple approach. These results potentially impact infant health outcomes by detecting key microorganisms to decipher fundamental trophic interactions in the human gut microbiome. We demonstrated the potential of pairwise assays using an unconventional method to provide insights into the emergent behaviors of the two strains in the presence of each other. These experiments suggest that cooperation between strains may expand HMO acquisition capabilities and help shift bacterial communities closer to those observed in a healthy infant gut microbiome.

## DECLARATIONS

### Copyright

© The Author(s) 2024.
