# Supplementary material for: Screening competition and cross-feeding interactions during utilization of human milk oligosaccharides by gut microbes
Source: Microbiome Res Rep. 2024 Jan 1;3(1):12. doi: 10.20517/mrr.2023.61 (PMC10917614; doi:10.20517/mrr.2023.61)
Supplement: Supplementary file 1 [file mrr-3-1-12-SupplementaryMaterials.pdf]

Zhang-Mills-Block - Modified Media (mZMB)

The mZMB medium consisted of 13 chemical groups. The following protocol was used to prepare 13 stock solutions for these groups, followed by mixing the stock solutions to prepare the final mZMB media.

- The concentrations of the chemical components were referenced from "Development of Chemically Defined Media Supporting High Cell Density Growth of Lactococci, Enterococci, and Streptococci"- Zhang, G. Appl Environ Microbiol. **2009**.
- Syringe-filter sterilization (SFS) was performed using 0.22um, pore size, Millex GP filter unit from Millipore.
- All stock solutions were transferred to 50 mL Falcon tubes before storage at -20 ºC.
- The chemicals were dissolved in 125 mL flasks prior to transfer to 50 mL Falcon tubes.
- All stock solutions were 50 mL except for Grp8, Vitamin: myo-inositol, which was 100 mL.

1. Grp8- Vitamin: myo-Inositol (0.1 g/L).

I. Add 10 mg of myo-inositol to a flask with a stir-bar.
II. Then, 30 mL of H_2O was added to the flask and the CS until dissolution.
III. Raise volume, with H_2O, to 100 mL.
IV. Store at -20 ºC.

2. Grp9- Phosphate buffers: KH_2PO_4 (52.4 g/L), K_2HPO_4 (107.1 g/L).

I. Add 2.6 g KH_2PO_4 and 5.4 g of K_2HPO_4 to the flask with a stir bar.
II. Add 30 mL of H_2O to the bottle and stir it (by hand) until dissolution.
III. Raise the volume, with H_2O, to 50 mL.
IV. Store at -20 ºC.

3. Grp11- Important Vitamin group: Calcium pantothenate (0.1 g/L), Niacin (0.075 g/L), Pyridoxal HCl (0.4 g/L).

I. Make (1.0 g/L) solution of calcium pantothenate by initially adding 50 mg of calcium pantothenate and 30 mL of H_2O into a separate 125 mL flask; CS was dissolved. Raise the volume, with H_2O, to 50 mL.
II. Make (0.75 g/L) was prepared by initially adding niacin (37.5 mg Niacin and 30 mL H_2O into a separate 125 mL flask; CS was dissolved. Raise the volume, with H_2O, to 50 mL.
III. In a new 125 mL flask with a stir bar, 20 mg of Pyridoxal HCl was added.
IV. Five mL of (1.0 g/L) Calcium pantothenate solution (5 mL of (0.75 g/L) Niacin solution (5 mL) were added.
V. Subsequently, 20 mL of H_2O was added to the flask and CS until dissolution.
VI. Raise the volume, with H_2O, to 50 mL.
VII. Store at -20 ºC.

4. Grp12- Important minerals group: $MgSO_4 \cdot 7H_2O$ (50 g/L), $FeSO_4 \cdot 7H_2O$ (0.2 g/L), $ZnSO_4 \cdot 7H_2O$ (0.25 g/L).

I. Add 2.5 g) was $MgSO_4$ to the flask with a stirring bar.
II. Add 30 mL of H_2O to the flask; CS.
III. Add 10mg of $FeSO_4$; CS.
IV. Add 12.5mg of $ZnSO_4$; CS until dissolved.
V. Raise the volume, with H_2O, to 50 mL.
VIII. Store at -20 ºC.

5. Grp13- Other Vitamin group: Folic acid (0.075 g/L), p-Aminobenzoic acid (0.0075 g/L)

I. Make (0.75 g/L) solution of Folic acid by initially adding 37.5 mg of folic acid and 30 mL of H2O into a separate 125 mL flask. Then, 200 uL of 1N NaOH; CS were added until dissolution. Raise the volume, with H_2O, to 50 mL.
II. Make (0.75 g/L) solution of p-Aminobenzoic acid by initially adding 37.5 mg of p-aminobenzoic acid and 30 mL of H_2O into a separate 125 mL flask; CS was dissolved. Raise the volume, with H_2O, to 50 mL.
III. Add 5 mL of (0.75 g/L) Folic acid solution and 500 uL of (0.75 g/L) p-Aminobenzoic acid solution to a new 125 mL flask.
IV. Raise the volume with H_2O to 50 mL and stir it by hand.
IX. Store at -20 ºC.

6. Grp14- Fatty acid group: Potassium acetate (90.1 g/L), Lipoic acid aka Thioctic acid (0.1 g/L), Tween 80 (50 g/L)

I. Make (1.0 g/L) solution of thioictic acid by initially adding 50 mg of thioictic acid and 30 mL of H_2O into a separate 125 mL flask. Add 500 µL of 1N NaOH; CS until dissolution. Raise the volume, with H_2O, to 50 mL.
II. Add 2.5 g of Tween 80 to the flask with a stirring bar.
III. Then, 30 mL H_2O was added to the flask.
IV. Stir the solution over the heating plate at low heat until dissolution. Heat was quickly removed once it dissolved.
V. Cool solution for approximately 5 min.
VI. Add 4.5 g of potassium acetate; CS.
VII. Thoictic acid solution (5 mL (1.0 g/L) was added to a new 125 mL flask.
VIII. Raise the volume, with H_2O, to 50 mL.
X. Store at -20 ºC.

7. Grp15- Nucleic acid base group: Adenine (1.5 g/L), Guanine (0.75 g/L), Uracil (3 g/L), Xanthine (0.5 g/L)

I. Add 75 mg adenine to the flask with a stir bar.
II. Add 20 mL of H_2O to the flask; CS.
III. Add 600 uL of 1N NaOH, drop by drop; CS until the precipitate is dissolved.
IV. Add 37.5 mg of Guanine; CS.
V. Add 1.9 mL of 1N NaOH, drop by drop; CS until the precipitate is dissolved.
VI. Add 150 mg of Uracil; CS.
VII. Xanthine (25 mg); CS were added.
VIII. Subsequently, 500 uL of 1N NaOH was added dropwise until the precipitate was dissolved.
IX. Raise the volume, with H_2O, to 50 mL.
XI. Store at -20 ºC.

8. Grp17- Traced mineral group: $(NH_4)6Mo7O_{24}\cdot 4H_2O$ (0.025 g/L), $MnSO_4\cdot 4H_2O$ (0.05 g/L), $CaCl_2\cdot 2H_2O$ (5 g/L), $CoSO_4\cdot 6H_2O$ (0.03 g/L), $CuSO_4\cdot 5H_2O$ (0.025 g/L), H_3BO_3 (0.1 g/L), K_2SO_4 (3 g/L), KI (0.015 g/L).

I. Make (0.25 g/L) solution of $(NH_4)6Mo7O_{24}\cdot 4H_2O$ by initially adding 12.5 mg $(NH_4)6Mo7O_{24}\cdot 4H_2O$ and 30 mL of H_2O into a separate 125 mL flask; CS was dissolved. Raise the volume, with H_2O, to 50 mL.
II. Make (0.5 g/L) solution of $MnSO_4\cdot 4H_2O$ by initially adding 25 mg of $MnSO_4\cdot 4H_2O$ and 30 mL of H_2O into a separate 125 mL flask; CS was dissolved. Raise the volume, with H_2O, to 50 mL.
III. Make (0.3 g/L) solution of $CoSO_4\cdot 6H_2O$ by initially adding 15 mg of $CoSO_4.6H_2O$ and 30 mL of H_2O into a separate 125 mL flask; CS was dissolved. Raise the volume, with H_2O, to 50 mL.

IV. Make (0.25 g/L) solution of $CuSO_4 \cdot 5H_2O$ by initially adding 15 mg of $CuSO_4 \cdot 5H_2O$ and 30 mL of H_2O into a separate 125 mL flask; CS until dissolved. Raise the volume, with H_2O, to 50 mL.

V. Make (1.0 g/L) solution of H_3BO_3 by initially adding 50 mg of H_3BO_3 and 30 mL of H_2O into a separate 125 mL flask; CS. Raise the volume, with H_2O, to 50 mL.

VI. Make (1.5 g/L) solution of KI by initially adding 75mg of KI and 30 mL of H_2O into a separate 125 mL flask; CS until dissolved. Raise the volume with H_2O to 50 mL; CS.

VII. In a new 125 mL flask with stir-bar, Add 250mg of $CaCl_2.2H_2O$.

VIII. Add 5 mL of (0.25 g/L) $(NH_4)6Mo7O_{24} \cdot 4H_2O$ solution, 5 mL of (0.5 g/L) $MnSO_4 \cdot 4H_2O$ solution, 5 mL of (0.3 g/L) CoSO4.6H2O solution, 5 mL of (0.25 g/L) $CuSO4 \cdot 5H_2O$ solution, 5 mL of (1.0 g/L) H_3BO_3 solution, and 500 uL of (0.15 g/L) KI solution.

IX. The solution was stirred until dissolution.

X. Add150mg of K_2SO_4; CS.

XI. Raise the volume, with H_2O, to 50 mL.

XII. Store at -20 ºC.

9. Grp18- Chelator group: EDTA (1.0 g/L), NTA aka Nitriliotriacetic acid (1.0 g/L).

I. Add 50 mg of EDTA to a flask in a beaker.

II. Add 10 mL of H2O to the flask; CS.

III. Add 50mg of NTA; CS.

IV. Add 500 µL of 1N NaOH; CS until dissolution.

V. Raise the volume, with H_2O, to 50 mL.

VI. Store at -20 ºC.

10. Grp19- Other component group- Glutathione (0.75 g/L), $(NH_4)2SO_4$ (50 g/L), NaCl (150 g/L).

I. Glutathione (37.5 mg) was added to a flask in a beaker.

II. Add 30 mL of H*2*O to the flask and add CS.

III. Add 2.5 g $(NH_4)2SO_4$; CS.

VII. Add 7.5 g of NaCl; CS until dissolved.

IV. Raise the volume, with H2O, to 50 mL.

V. Store at -20 ºC.

11. Grp21- EAA#8: Biotin (0.5 g/L).

I. Biotin (25 mg) was added to the flask with a stir bar.

II. Add 500 uL of 1N NaOH and 4 mL of H_2O; Stir until dissolved.

III. Raise the volume with H_2O to 50 mL; CS.

VI. Store at -20 ºC.

12. Grp22- EAA#9: Thiamine HCl (0.075 g/L).

I. Make (0.75 g/L) solution of Thiamine HCl by initially adding Thiamine HCl (37.5 mg Thiamine HCl and 30 mL H_2O into a separate 125 mL flask; CS until dissolved. Raise the volume, with H_2O, to 50 mL.

II. In a new 125 mL flask, Add 5 mL of (0.75 g/L) Thiamine HCl solution.

III. Raise the volume, with H_2O, to 50 mL.

IV. Store at -20 ºC.

13. Grp23- EAA#10: Riboflavin (0.075 g/L)

I. Make (0.75 g/L) solution was prepared by initially adding riboflavin (37.5 mg Riboflavin and 30 mL H_2O into a separate 125 mL flask. Add 600 uL of 1N NaOH; CS until dissolved. Raise the volume, with H_2O, to 50 mL.

II. In a new 125 mL flask, Add 5 mL of (0.75 g/L) Riboflavin solution.

III. Raise the volume, with H_2O, to 50 mL.
IV. Store at -20 ºC.

100 mL of 1N NaOH:

I. Then, 70 mL H_2O was added to the beaker. The beaker was then placed in a bucket of ice.
II. Add 4 g of NaOH slowly.
III. Stir until dissolved.
IV. The solution was then poured into a glass graduated cylinder.
V. The final volume was increased to 100 mL using H_2O.
VI. The final solution was poured into a new 500 mL bottle.
VII. Store at room temp.

100 mL of 10N NaOH:

I. Then, 70 mL H_2O was added to the beaker. The beaker was then placed in a bucket of ice.
II. Then, 40 g of NaOH was slowly added.
III. Stir until dissolved.
IV. The solution was then poured into a glass graduated cylinder.
V. The final volume was increased to 100 mL using H_2O.
VI. The final solution was poured into a new 500 mL bottle.
VII. Store at room temp.

To prepare 50 mL of mZMB medium,

I. Added in descending order of group numbers:

Group no.	Volume (mL) add	Group no.	Volume (mL) add	Group no.	Volume (mL) add
**8**	1	**13**	0.373	**19**	1
**9**	3	**14**	0.5	**21**	0.6
**11**	0.6	**15**	0.367	**22**	0.373
**12**	1	**17**	0.38	**23**	0.6
		**18**	0.375		

II. The volume of the media was 23.9 mL after adding group #23. Next, add 16.1 mL of H_2O was added and stirred using a magnetic stirrer bar. The resulting volume was 40 mL, with a pH of 6.76.
III. During stirring, 100 uL of 10N NaOH at a time, drop by drop, to adjust the final pH to 7.8. Amount of 10N NaOH used was varied.
IV. H_2O was then added to increase the final volume to 50 mL. The final volume of H_2O may vary depending on the amount of NaOH used to adjust the pH.
V. SFS into a sterile 50 mL falcon tube.
VI. Covered in aluminum foil.
VII. Store at 4 ºC.
